# Complex Links between Natural Tuberculosis and Porcine Circovirus Type 2 Infection in Wild Boar

**DOI:** 10.1155/2014/765715

**Published:** 2014-06-03

**Authors:** Iratxe Díez-Delgado, Mariana Boadella, MariPaz Martín-Hernando, José Angel Barasona, Beatriz Beltrán-Beck, David González-Barrio, Marina Sibila, Joaquín Vicente, Joseba M. Garrido, Joaquim Segalés, Christian Gortazar

**Affiliations:** ^1^Departamento de Sanidad Animal, Facultad de Veterinaria, Universidad Complutense de Madrid, 28040 Madrid, Spain; ^2^SaBio-IREC (CSIC-UCLM-JCCM), Ronda de Toledo s/n, 13005 Ciudad Real, Spain; ^3^Centre de Recerca en Sanitat Animal (CReSA), UAB-IRTA, Campus de la Universitat Autònoma de Barcelona, 08193 Bellaterra, Spain; ^4^Instituto Vasco de Investigación y Desarrollo Agrario (NEIKER), Departamento de Sanidad Animal, Berreaga 1, Derio, 48160 Vizcaya, Spain; ^5^Departament de Sanitat i d'Anatomia Animals, Universitat Autònoma de Barcelona, 08193 Bellaterra, Spain

## Abstract

Individuals in natural populations are exposed to a diversity of pathogens which results in coinfections. The aim of this study was to investigate the relation between natural infection with tuberculosis (TB) due to infection by bacteria of the *Mycobacterium tuberculosis* complex and porcine circovirus type 2 (PCV2) in free-ranging Eurasian wild boar (*Sus scrofa*). Apparent prevalence for TB lesions and PCV2 infection was extremely high in all age classes, including piglets (51% for TB; 85.7% for PCV2). Modeling results revealed that the relative risk of young (less than 2 years old) wild boar to test positive to PCV2 PCR was negatively associated with TB lesion presence. Also, an interaction between TB, PCV2, and body condition was evidenced: in wild boar with TB lesions probability of being PCV2 PCR positive increased with body condition, whereas this relation was negative for wild boar without TB lesions. This study provides insight into the coinfections occurring in free-ranging host populations that are naturally exposed to several pathogens at an early age. Using TB and PCV2 as a case study, we showed that coinfection is a frequent event among natural populations that takes place early in life with complex effects on the infections and the hosts.

## 1. Introduction

As opposed to controlled laboratory environments, individuals in natural populations are exposed to a diversity of pathogens (viruses, bacteria, and parasites) which results in coinfections [1]. Each pathogen interacts with the host immune system, generating synergy or antagonism with other pathogens [2]. This has important implications both for the host [3, 4] and the pathogens [5–8]. In the last decade, infectious disease research has shifted from traditional one host–one pathogen approaches to multihost–multipathogen approaches, often incorporating concepts and techniques from community ecology [3, 4, 9]. The community ecology approach is useful for coinfection studies because pathogens interact by competing for resources (bottom-up strategies) or through modifications in the host immune system (top-down strategies) [3].

Regarding synergies among pathogens there are some well-documented cases. For instance, due to its effect on the immune system, the human immunodeficiency virus (HIV) increases the risk of malaria infections [10] and facilitates infection by* Mycobacterium tuberculosis *(*M. tuberculosis*)[11], which causes human tuberculosis (TB). The interaction is reciprocal, since TB, in turn, is known to promote the course of HIV [12]. Increased host susceptibility to* Mycobacterium bovis *(*M. bovis*), the main causative agent of animal TB, has been suggested in helminth infected African buffalo (*Syncerus caffer*) due to a tradeoff in the type of immune response [13]. Other coinfections associated with more severe diseases or increased mortality include* Sarcocystis neurona* and* Toxoplasma gondii *in marine mammals [14] and* Babesia* spp. and Canine Distemper virus (CDV) in Serengeti lions [15]. However, no such synergy was evidenced in lions coinfected with feline immunodeficiency virus (FIV) and* M. bovis* [16].

Antagonism, meaning decrease of host susceptibility or pathogen fitness, has been documented between different parasites in several species [17–21] and has also been described in humans coinfected with HIV and GB virus type C (GBV-C) [22, 23], a common nonpathogenic human flavivirus formerly known as hepatitis G virus.

While human TB caused by* M. tuberculosis *has monopolized all the attention, zoonotic TB due to* M. bovis* has been neglected and now represents an important reemerging public health issue in developing countries [24, 25]. In developed countries, where* M. bovis* is subjected to disease control measures in cattle, wildlife reservoirs are becoming an increasing concern regarding their role in infection maintenance [26]. The native Eurasian wild boar has been identified as a key wildlife maintenance host of* M. bovis* and other closely related members of the* M. tuberculosis* complex (MTC) [26, 27]. TB monitoring and several attempts to reduce infection prevalence in this host are ongoing (e.g., [28–30]). In this context, it has been postulated that coinfection with PCV2 may increase wild boar population susceptibility to MTC infection, potentially interfering with TB control [31].

Porcine circovirus type 2 (PCV2) is an ubiquitous and easily transmitted virus, considered the etiological agent of a number of syndromes known as porcine circovirus diseases [32]. The most relevant one is PCV2-systemic disease (PCV-SD), formerly known as postweaning multisystemic wasting syndrome (PMWS), an immunosuppressing disease of late nursery and fattening domestic pigs [32, 33]. Coinfection with other pathogens is commonly reported in PCV2-SD affected pigs and it is suggested that the acquired immunodeficiency developed by diseased animals predisposes to coinfections [33]. This syndrome has also been described in Eurasian wild boar (*Sus scrofa*) piglets [34, 35]. In Spain, PCV2 seroprevalence in wild boar has been reported to be around 48% [36]. This prevalence has increased in some fenced wild boar populations (those managed for hunting purposes) during the last decade [29]. It has also been postulated that PCV2-SD might play a role in the population dynamics of intensively managed (fenced and artificially fed) wild boar populations, through increased piglet mortality [36].

The aim of this study was to investigate the relation between natural infection with tuberculosis (TB) due to infection by bacteria of the* Mycobacterium tuberculosis* complex and porcine circovirus type 2 (PCV2) in free-ranging Eurasian wild boar (*Sus scrofa*).

## 2. Materials and Methods

### 2.1. Study Area

Sampling sites included private and public hunting estates (*n* = 14) of Montes de Toledo (39°25′ to 39°16′N, 4°05′ to 4°23′W), a mountain chain located in the South of the Central Spanish plateau. Study sites are mainly devoted to recreational hunting for wild boar and red deer (*Cervus elaphus*) and represent a gradient of management situations [37].

### 2.2. Sampling and Data Collection

Samples were collected from 216 wild boars harvested during the 2011-2012 hunting season (October–February). After each hunting event, a representative sample stratified by age and sex of the hunted animals was randomly selected. Each specimen was subjected to a quick general inspection, collection of biometrical data, and sex and age determination. Age class estimation was done based on tooth eruption patterns [38]: wild boars less than 12 months old were classified as piglets (*n* = 49), those between 12 and 24 months as subadults (*n* = 55), and those over 2 years as adults (*n* = 112). We grouped animals less than 2 years (piglets and subadults) as young wild boar.

Mandibular lymph nodes (LNs), tonsils, tracheobronchial LN, mediastinal LN, lungs, spleen, and mesenteric LN, as well as other affected organs were collected during field inspection and then subjected to detailed inspection in the laboratory and stored at −20°C. An extra copy of the tissues of 67 young wild boars was also fixed by immersion in 10% neutral-buffered formalin for histopathology and* in situ* hybridization (ISH) studies. Serum was obtained after centrifugation of blood samples obtained from the thoracic cavity and stored frozen at −20°C until use.

### 2.3. Pathology, Culture, Serology, and PCR

The detection of TB-compatible lesions is a good proxy for MTC infection in wild boar [39, 40]; therefore, this variable was systematically recorded for all 216 studied wild boars, following a protocol that included the inspection of all relevant organs [40]. Additionally, TB compatible lesions were scored as 0 (no visible lesions), 1 (lesion diameter < 10 mm), or 2 (at least one lesion > 10 mm). Taking into account all examined organs (6 LN, including left and right mandibular, left and right tracheobronchial, mediastinal and mesenteric, and the 7 lung lobes separately), the total TB lesion score of an individual potentially ranged from 0 to 26.

Tissue samples (mandibular LN and tonsil pool plus a thoracic LN pool) of 59 young wild boars were cultured following the procedures described in Garrido et al. [41]. All isolates were spoligotyped in order to confirm the strain [42]. Any individual wild boar with a mycobacterial growth confirmed by spoligotyping as belonging to the MTC was defined as culture-positive.

Taking into account that PCV2 causes disease mainly in younger age classes, haematoxylin-eosin stained slides (4 µ thick) of 67 young wild boars (25 piglets and 42 subadults) were examined for assessing the presence or absence of lymphoid depletion in spleen and LN tissue and for microscopic TB lesions in mandibular and thoracic LNs as well as the lung [43]. Additionally, a previously described ISH technique [44] was performed on a subsample (*n* = 19) of formalin-fixed, paraffin-embedded tissue samples corresponding to 6 piglets and 13 subadults.

The assessment of PCV2 serological status was made by means of a commercial indirect ELISA (INGEZIM CIRCO IgG, INGENASA, Madrid, Spain). Following manufacturer instructions, the positive cut-off was calculated as the mean OD of negative controls + 0.25.

Extracted DNA (DNeasy extraction kit, Qiagen GmbH, Germany) from lung tissue samples was processed by standard PCV2 PCR [45].

Due to funding and logistic limitations not all wild boar could be submitted for* M. bovis* culturing and PVC2 PCR and ISH. The subsets submitted for these tests were composed by randomly selected wild boars. Sample size (sorted by age class) used in each diagnostic method is summarized in Table 1.

### 2.4. Statistical Methods

Sterne's exact method was used to estimate the apparent prevalence with 95% confidence intervals (CIs). Comparison of mean TB-compatible lesion score between young age classes (piglets and subadults) was analyzed by means of Mann-Whitney's* U* test.

Factors related with TB and PCV2 coinfection (probability of being TB-lesion or PCV2 PCR positive) were studied in the subset of 66 young wild boars (24 piglets and 42 subadults) by means of 2 generalized mixed linear models (GzLMM): (1) a model to assess the probability of being TB-lesion positive (TB model) and (2) a second one to assess the probability of being positive to PCV2 PCR (PCV2 model). Both models were built with a binomial structure and a logit link function including hunting estate as a random factor. Common explanatory variables for both models were sex (male or female), age (piglets or subadults), body condition (estimated as thoracic perimeter/total body length and categorized as low or high using the median value as a cut-off point), and lymphoid depletion (presence/absence); 2-way interactions between all variables were also included. Collinear explanatory variables were excluded from the model according to Hosmer and Lemeshow 2000 by using Spearman's pairwise correlation coefficient |*r* | >0.3. The TB model used PCV2 PCR results as an explanatory variable and vice versa. The *P*-value was set at 0.05. Data was analyzed using IBM SPSS statistical package version 20 (IBM Corporation, Somar, NY, USA).

## 3. Results

**Figure 1:**
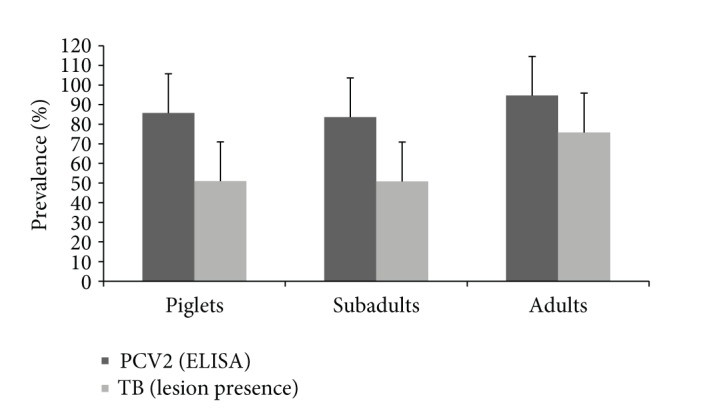
Mean prevalence for presence of tuberculosis (TB) compatible lesions and porcine circovirus type 2 (PCV2) antibody prevalence by age class. Error bars indicate 95% CI.

Figure 1 shows the results of the initial screening of the 216 wild boars of all age classes for TB-compatible lesions and for antibodies against PCV2. Apparent prevalence for both was high in all age classes, including piglets (51% TB-compatible lesion prevalence; 85.7% PCV2 antibody prevalence). Regarding wild boar with TB-compatible lesions, the mean TB lesion score was higher for piglets (4.29 ± 0.72) than for subadults (3.71 ± 0.83), although the difference between mean values was not significant (*Z* = −1.302, *P* > 0.05). The mean lesion score in adult wild boar was 5.13 ± 0.510.

MTC infection was confirmed by culture in 31 (52.5%) young wild boar (52.4% in piglets and 52.6% in subadults). The kappa agreement between presence of TB-compatible lesion and MTC culture was substantial (0.66 at 95% CI; *n* = 59). Of the 67 young wild boars submitted for histopathology, 80.6% displayed a slight degree of lymphoid depletion. PCR for PCV2 yielded positive in 30 (45.5%) young wild boars (with prevalence ranging from 25% in piglets to 57.1% in subadults). From the 19 animals analyzed by ISH, 5 (26.3%) resulted positive. Two of them were positive both in the lung and LN sections and three were positive only in the LN. These five ISH positive wild boars also displayed a slight degree of lymphoid depletion.

**Figure 2:**
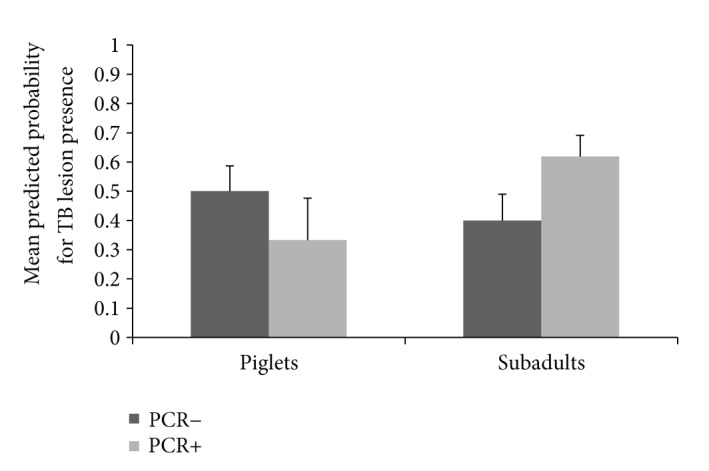
Relationship between the predicted probability for tuberculosis (TB) lesion presence and porcine circovirus type 2 (PCV2) PCR results according to age class (bars represent 95% CI).

**Figure 3:**
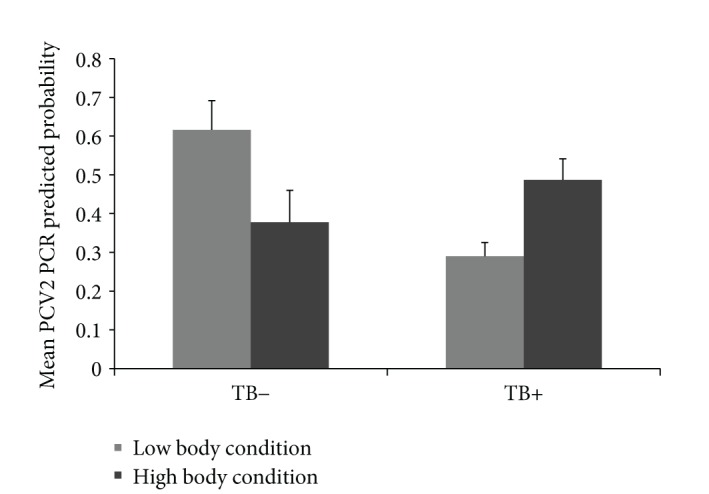
Relationships between porcine circovirus type 2 (PCV2) PCR results and body condition (categorized as low or high using median as a cut-off point) according to tuberculosis (TB) lesion absence or presence (bars represent 95% CI).

Results of the two GzLMMs are displayed in Table 2. In the TB model, no statistical differences were evidenced between age classes. However, the interaction between age class and PCV2 PCR resulted in a statistically significant factor. The probability of presenting TB lesions was lower for PCV2 PCR positive piglets but higher for PCV2 PCR positive subadults (*Z* = −1.98, *P* < 0.05, Figure 2). In the PCV2 model, no statistical differences were evidenced between age classes. However, the relative risk to test positive to PCV2 PCR was negatively associated with TB lesion presence regardless of age. Also, an interaction between TB, PCV2, and body condition was evidenced. In wild boar with TB lesions the probability of being PCV2 PCR positive increased with body condition, whereas this relation was negative for wild boar without TB lesions (Figure 3).

## 4. Discussion

This study provides insight into the coinfections occurring in free-ranging host populations that are naturally exposed to several pathogens. Using MTC and PCV2 as a case study, we showed that coinfection is a frequent event among natural populations that takes place early in life with complex effects on the hosts and outcome of infection, acting as a risk factor.

This study revealed very high infection prevalence for both MTC (52%) and PCV2 (45%) in naturally exposed young wild boars. Regarding TB, Vicente et al. [40] found about 43% of TB-compatible lesions in this age class. Respecting PCV2 actual prevalence figures are likely even higher, considering the recorded antibody prevalence of 85%. This implies that wild boars from the study region are exposed early in life to an extremely high infection pressure. For comparison, Vicente et al. [36] recorded 38% antibody prevalence against PCV2 in fenced wild boar piglets, and Boadella et al. [29] found 47%. Regarding coinfection data on these pathogens and Risco et al. [46] found correlations between PCV2 infection prevalence and TB prevalence in wild boar in other high TB and PCV2 prevalence sites in Mediterranean Spain. By contrast, both PCV2 and TB have a much lower prevalence (<10%) in Spanish regions with a more humid Atlantic climate where, interestingly, generalized TB appears to be less common [37, 47]. Lymphoid depletion, a likely consequence of clinical PCV2 infection, is not the only mechanism through which PCV2 modulates immune function as cytokines and innate immune system play also a role in its pathogenesis [33]. There are several studies which show that an established immune profile against a given infection could affect the outcome of subsequent infections or vaccinations [17, 48–50]. Since evidence of a dichotomy in the humoral and cellular immune responses exists in pigs [51], we suggest that early PCV2 infection somehow impairs the ability of wild boar piglets to respond to other infections, including MTC.

However, the specific relations between PCV2 positivity by PCR and presence of TB-compatible lesions are not easy to interpret with the data obtained in this observational study. Some rather counterintuitive findings include the negative association between both infections and the higher TB score in piglets. Wild boar piglets in this population could suffer an increased mortality if coinfected with PCV2 and MTC. Some of these individuals could be lost from the population before being sampled (during the hunting season, October to February), contributing to the observed age by infection interaction [36].

Finally, the interaction between TB, PCV2, and body condition was also counterintuitive, since wild boar with TB lesions had a positive relation between PCV2 PCR and body condition, whereas this relation was negative for wild boar without TB lesions. Subclinical PCV2 infection does not negatively affect the body condition [52]. It is also unlikely that TB-lesion presence* per se* had such an effect. Unpublished data from this study area show no evidence of poor body condition in TB-lesion positive wild boar. It is well known that management practices that promote aggregation of wild boar, such as supplementary feeding, are associated with higher infection risks by bacterial and viral pathogens including MTC and PCV2 (e.g., [37, 53]). Thus, we propose that in the studied population, aggregation due to supplementary feeding could be the mechanism linking a high body condition to a high probability of coinfection. Supplementary fed wild boar would be in better condition, but at the same time, more likely to contact MTC and PCV2 through spatial aggregation at feeders. By contrast, a low body condition would be associated with a lower chance of coinfection because of the lower spatial aggregation if no feeding takes place, but perhaps also because coinfected animals with a poor body condition are less likely to survive (and therefore, to be sampled).

We demonstrated that coinfection is a frequent event in this wild boar population, but the nature of this interaction was more complex than expected. Moreover, there are many more coinfections likely to be occurring at the same time. In the present study, two relevant pathogens were analyzed, but part of the findings may be due to other (unknown) infections and interactions taking place at the same time, in a much more complex multipathogen interaction network. Furthermore, this cross-sectional study is based on time point data and therefore the infection sequence cannot be established. In some cases timing is important in regard to the coinfection outcome, as experimental coinfection trials reveal: for instance, previous infection with* M. hyopneumoniae* potentiates severity of PCV2 disease in pigs [54–56] but no effects on clinical signs and pathology are observed in simultaneous coinoculation [57]. We ignore if this occurs in the wild boar, and similar experimental studies in this host would be advisable.

In humans, the bidirectional interaction between the virus HIV and TB is well documented [11, 12] (each of them acts as a risk factor to the other, by favoring infection or accelerating progression). In wild boar, PCV2 and TB coinfection could make a similar picture due to the resembling characteristics of these pathogens to HIV-TB. Since PCV2 is a well-known agent able to modulate the immune response [33], it is likely that it may induce a certain degree of immune-compromise in this highly prevalent wild boar population. Such scenario may facilitate interaction with other pathogens, a fact that should be taken into account when dealing with the epidemiology of natural infections. Globally, this situation may have implications regarding the assessment and even the efficacy of future disease control measures [46, 58]. This confirms the view that in natural settings multipathogen approaches are more realistic than single-pathogen ones. Further investigation of the pathogenesis of MTC/PCV2 coinfection is advisable, particularly regarding the fact that PCV2 infection might have an effect on the ongoing TB control efforts through oral vaccination of wild boar [28]. If confirmed, this finding would have implications regarding the likelihood of coinfected individuals to become significant TB excretors (individuals that contribute disproportionately to disease transmission [59]) or even the response of PCV2 infected wild boar piglets to vaccination (but see [50]).

## 5. Conclusions

Summarizing, this study evidences that coinfection is a frequent event in free-ranging populations and may have complex effects on the infections and the hosts, acting as a risk factor specially when prevalence is high and any of the pathogens has an immunosuppressive feature. Moreover, in a TB control integrated strategy scenario coinfections might have an implication on the outcome of these measures.

## Figures and Tables

**Table 1:** Eurasian wild boar (*Sus scrofa*) available for porcine circovirus type 2 (PCV2) and tuberculosis (TB) status assessment in Montes de Toledo, Spain.

Age class	PCV2 status assessment				TB status assessment	
	iELISA	Lymphoid depletion	PCR	ISH	Lesions and score	Culture
Piglets	49	25	24	6	49	21
Subadults	55	42	42	13	55	38
Adults	112	—	—	—	112	—

Total	216	67	66	19	216	59

**Table 2:** Results of the two GzLMMs to identify drivers of TB lesion presence and PCV2 PCR positivity. The models were fitted using hunting estate as a random factor, a binomial distribution, and a logit link. Parameter estimates (*β*) for the level of fixed factors were calculated using a reference value of 0 for the “female” and “subadult” levels in the explanatory variables sex and age class, respectively. Significant *P* values are shown in bold.

Explanatory variable	DF	*F*-value	*P*	*β*
TB lesion presence due to PCV2 co-infection model				
Sex	1,45	0	0.997	Males = 0.432
Age class	1,45	0	0.991	Piglets = −3.008
Sex ∗ age class	1,45	0.541	0.466	1.343
Body condition	1,45	0	0.983	−17.768
Lymphoid depletion	1,45	0	0.984	Negative = 483.743
PCV2 PCR	1,45	3.238	0.079	Negative = −22.051
Sex ∗ lymphoid depletion	1,45	0	0.997	10.192
Age class ∗ lymphoid depletion	1,45	0	0.991	−40.206
Sex ∗PCV2 PCR	1,45	0.479	0.493	−1.277
Age class ∗ PCV2 PCR	1,45	4.311	**0.044**	**4.170**
Body condition ∗ lymphoid depletion	1,45	0	0.983	−636.392
Body condition ∗ PCV2 PCR	1,45	3.173	0.082	26.055

PCV2 PCR positivity due to TB coinfection model				
Sex	1,45	0.770	0.385	Male = 1.394
Age class	1,45	0.012	0.914	Piglet = 0.506
Sex ∗ age class	1,45	0.824	0.369	−1.289
Body condition	1,45	1.863	0.201	12.216
Lymphoid depletion	1,45	2.018	0.162	Negative = −23.261
TB lesion presence	**1,45**	**4.224**	**0.046**	**Negative = **−**21.430**
Sex ∗ lymphoid depletion	1,45	1.572	0.216	−3.257
Age class ∗ lymphoid depletion	1,45	1.445	0.236	2.503
Sex ∗ TB lesion presence	1,45	0.162	0.689	−0.578
Age class ∗ TB lesion presence	1,45	2.963	0.092	−2.470
Body condition ∗ lymphoid depletion	1,45	1.810	0.185	29.370
Body condition ∗ TB lesion presence	**1,45**	**4.176**	**0.047**	−**25.405**
